# A randomized controlled trial of a targeted support program for informal caregivers in adult psychiatry

**DOI:** 10.3389/fpsyt.2023.1284096

**Published:** 2023-11-30

**Authors:** Shyhrete Rexhaj, Debora Martinez, Philippe Golay, Claire Coloni-Terrapon, Shadya Monteiro, Leslie Buisson, Anne-Laure Drainville, Charles Bonsack, Alban Ismailaj, Alexandra Nguyen, Jérôme Favrod

**Affiliations:** ^1^La Source, School of Nursing Sciences, HES-SO University of Applied Sciences, Lausanne, Switzerland; ^2^Community Psychiatry Service, Department of Psychiatry, University Hospital, CHUV, University of Lausanne, Lausanne, Switzerland; ^3^Institute of Psychology, Faculty of Social and Political Science, University of Lausanne, Lausanne, Switzerland; ^4^SISP SA, Lausanne, Switzerland

**Keywords:** informal caregivers, mental health, randomized controlled trial, individual intervention, burden, painful emotions

## Abstract

**Background:**

The importance of informal caregivers for persons with severe mental illness has been demonstrated. However, this role may cause a high care burden that considerably affects caregiver health. The Ensemble program is a five-session brief individual intervention designed to support informal caregivers. This trial aimed to assess the efficacy of the program versus SAU (support as usual) for participants with a high care burden.

**Methods:**

A single-center randomized controlled trial including 149 participants was conducted. Caregivers in the intervention arm participated in the Ensemble program. The effects of the intervention were assessed using mixed models for repeated measures analysis of variance on improvements in informal caregivers’ psychological health status, optimism levels, burden scores, and quality of life at three time points (T0 = pretest; T1 = posttest at 2 months, and T2 = follow-up at 4 months).

**Results:**

Analysis of the Global Psychological Index showed no significant effect at the two endpoints in favor of the Ensemble group. However, the Brief Symptom Inventory-Positive Symptom Distress Index was significantly lower at the two-month follow-up. A significant reduction in burden on the Zarit Burden Interview was observed post-intervention, along with an increase in optimism levels on the Life Orientation Test-Revised at follow-up in the Ensemble group. No significant differences were observed in quality of life. Clinical improvements in both psychological health status and burden levels were also identified.

**Conclusion:**

The Ensemble program offers an inclusive approach based on a recovery perspective that significantly reduces symptom distress and burden and increases optimism among informal caregivers.

**Clinical trial registration**: https://clinicaltrials.gov/, NCT04020497.

## 1 Introduction

Informal caregivers play a vital role in the treatment and support of close relatives with severe and persistent mental illness (1). An informal caregiver of psychiatric patients is an individual who provides unpaid support and assistance to someone experiencing mental health challenges or psychiatric illnesses. These caregivers may be family members, close friends, or other loved ones (2). Informal caregivers often provide interventions such as emotional and social support, practical assistance in everyday activities, medication management, monitoring and observation of behavior and mood, coordination and help to navigate the healthcare system, attending appointments with healthcare professionals, and crisis and emergency interventions (3). The scientific literature reports considerable unmet needs among informal caregivers (4, 5), noting that relatives who provide care want further clarification of their roles and responsibilities, greater control over their lives, effective collaboration with health professionals, and tailored information, practical assistance, and emotional support (1, 5–7).

Interventions for families of patients with severe mental disorders are well-established but remain heterogeneous (8). Online programs have been effective in increasing caregivers’ knowledge and decreasing their perceived family stress and burden (9). Among existing face-to-face support programs, most are group interventions that may include the care recipient (10). These programs focus on building peer support, psychoeducation, improving family relationships, and promoting self-care, compassion, problem-solving skills, and communication (10, 11). Despite the growing development of such support interventions, only a few have focused on specific caregiver outcomes, such as burden or psychological distress, and offered support tailored to individual caregivers’ needs (7, 11–13). To meet the unique needs of each person, interventions should be adjusted to match the informal caregiver’s role, how they view the illness, how they cope, and the specific conditions within the healthcare system and environment (4, 14–19).

Ensemble is a targeted individual support intervention for informal caregivers of patients in adult psychiatry (16–70 years old). It has been tested in a pilot study and found to significantly improve psychological health status and optimism levels (2, 6). The Ensemble randomized controlled trial (RCT) presented in this study was a monocenter RCT designed to assess the efficacy of the Ensemble program. The program was administered in five individual sessions, utilizing different practical exercises and tools according to the needs of each participant, including the assessment of needs, problem-solving, and positive and assertive communication (20).

### 1.1 Aim of the trial

This trial aimed to assess the efficacy of the Ensemble program versus support as usual (SAU) in terms of caregivers’ psychological health status, optimism levels, burden, and quality of life at the end of the trial (2 months after baseline). It also aimed to evaluate the maintenance of the clinical effect at follow-up (4 months after baseline).

## 2 Methods

### 2.1 Hypothesis and design

The primary hypothesis proposed that compared with the SAU group, five one-hour sessions of the Ensemble program would lead to improved psychological health status, as evaluated by the Global Severity Index (GSI) of the Brief Symptom Inventory (BSI) (21). The BSI comprises 53 items organized into nine primary and clinically relevant symptom dimensions and three global severity indices. The secondary hypothesis proposed that the Ensemble program would increase informal caregivers’ optimism levels, measured by the Life Orientation Test-Revised (LOT-R) (22), decrease their burden score on the French version of the Zarit Burden Interview (ZBI) (23) and improve their quality of life, assessed by the 36-item Medical Outcome Study Short-Form Health Survey (SF-36) (24). The study also monitored the sustainability of potential benefits at follow-up (2 months after completing the Ensemble program). In addition to its statistical effects on the intervention group, the study explored clinical benefits on psychological health and burden compared with the SAU group.

### 2.2 Participants

This study was conducted in seven cantons (Vaud, Valais, Fribourg, Neuchâtel, Genève, Bern, and Jura) in French-speaking Switzerland. The study included informal caregivers and individuals providing close, informal support to adult patients with psychiatric disorders. Participants were required to meet the following inclusion criteria: being at least 18 years old, living in French-speaking Switzerland, speaking French, having an adult relative suffering from a psychiatric disorder (with or without an established diagnosis), and having the capacity to agree to participate in an Ensemble trial. The exclusion criterion was a score < 20 on the ZBI (23). To offer support to all informal caregivers according to their needs, it was sufficient if they considered themselves in need of support in their caregiving role. The Social and Occupational Functioning Assessment Scale (SOFAS) (25) was used at baseline to measure patients’ professional and social functioning levels, as reported by the participants with the assistance of a research assistant, if necessary. Participants were recruited from family associations and public mental health services in French-speaking Switzerland. Meetings were conducted and regular information was disseminated by general practitioners, local newspapers, schools, social and cultural centers, and social networks to ensure equivalent access to information for all informal caregivers, regardless of their situation. The research coordinator was directly accessible via email or telephone. General information about the project was also available online, and the research coordinator assessed primary inclusion criteria and ensured first contact between the assessor and participant.

### 2.3 Randomization

The randomization process was computer-controlled using Research Electronic Data Capture (REDCap) (26). The data manager created an Excel list for stratified group randomization and transferred it to the REDCap platform, which then automatically allocated all the participants to either the intervention or control group. Depending on the outcome of randomization, participants were referred to a professional conducting the intervention by a person not involved in the research.

### 2.4 Outcome assessment

The Ensemble trial involved three assessments (T0 = pretest, T1 = posttest at 2 months, and T2 = follow-up at 4 months) conducted by a trained research assistant. All outcomes were measured using scores from the validated French versions of the scales in the self-reported questionnaires, which were integrated into the electronic survey managed by REDCap software. All the self-reported scales were completed by the participants. If necessary, a research assistant answered any questions or clarified the items. In addition, the research assistant, who was blinded to the intervention allocation, provided technical assistance to participants and shared their experiences with them. After T2, the participants in the control group also received the benefits of the program.

### 2.5 Sample size

The expected effect size for the GSI difference between the two groups, which was obtained from the pilot study outcomes, was Cohen’s *d* = 0.470. With β set at 0.05 and a power of *β* =0.80, an *a priori* computation for ANCOVA indicated that the proposed trial required a total sample size of 144 participants across both arms, with 72 participants in each arm. A total of 160 participants were recruited, with a potential dropout rate of approximately 10%.

### 2.6 Ethical considerations

The Human Research Ethics Committee of Vaud State, Switzerland, approved the research protocol (Project ID-2019-01181). All individuals who agreed to participate signed a consent form and were interviewed individually. Written informed consent was obtained from all participants. The study was conducted in accordance with the recommendations of the Human Research Ethics Committee of Canton Vaud and the Declaration of Helsinki. No severe adverse event occurred during the study.

### 2.7 Interventions

#### 2.7.1 Support as usual

SAU comprises informal support for caregivers, offered by the patient’s clinical team. This support includes specific psychoeducational programs that are developed based on the patient’s illness, or peer support and voluntary efforts from family associations. In addition, general professional services are available in the study area to inform and orient informal caregivers or relatives if needed. Personal psychiatric treatment is also available for those in need of it.

#### 2.7.2 Ensemble program

**Figure 1:**
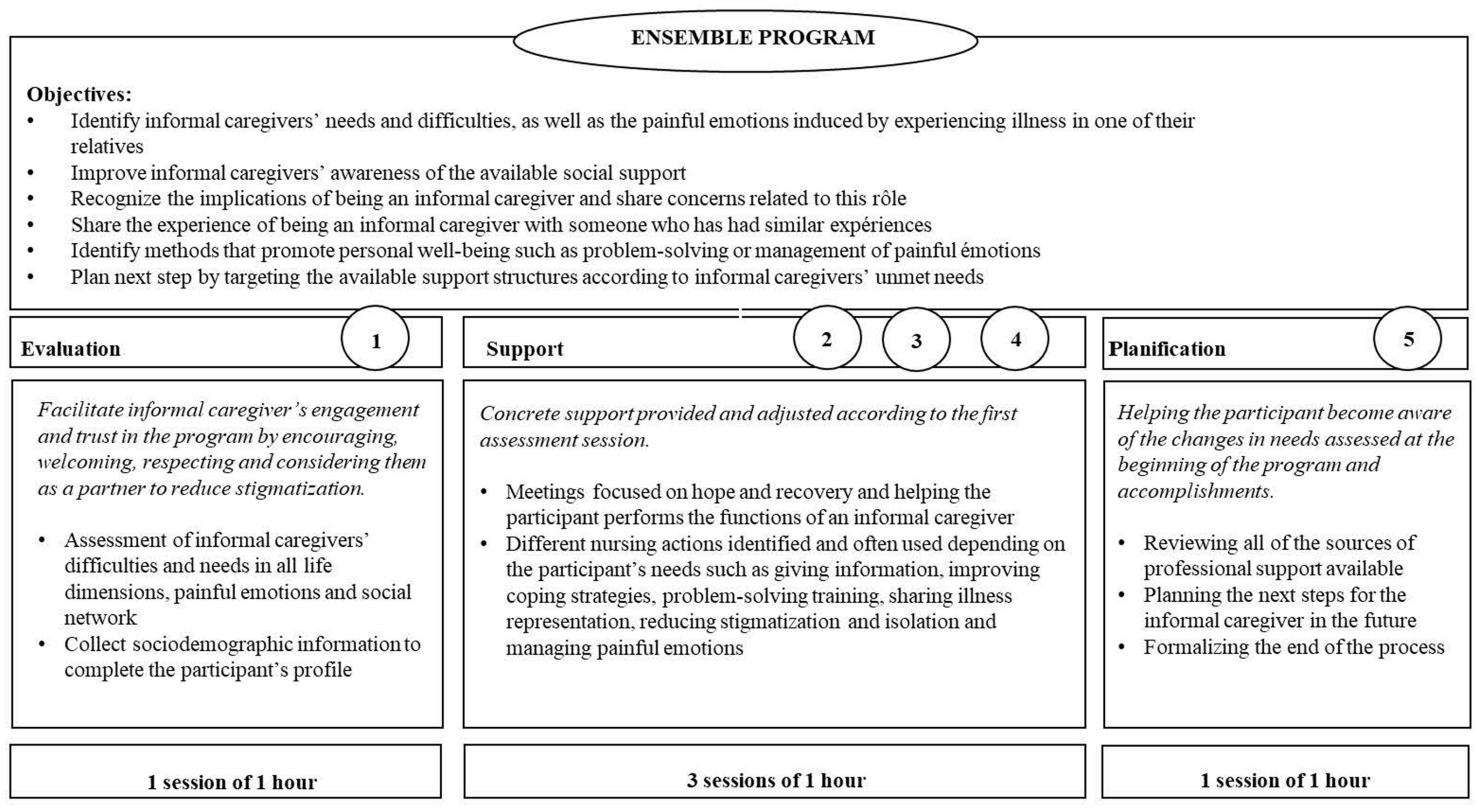
Ensemble program and process (19).

The Ensemble program addresses the specific unmet needs, painful emotions, and social resources of informal caregivers, and aims to adapt care activities for each participant (Figure 1). The intervention’s development and program content have been described in previous studies (6, 20). The program promotes informal caregivers’ well-being and encourages them to reflect on their caregiving role. It consists of a brief, individual, five-session process that provides targeted support for informal caregivers’ concerns. The first session concerned personal difficulties and needs across various life dimensions. Painful emotions and social resources were thoroughly evaluated. The participants were welcomed and engaged as active participants. Sessions 2, 3, and 4 focused on hope, recovery, illness, services, caregivers’ role involvement, emotional management, communication skills, problem-solving, and knowledge of stigmatization according to prioritized needs. The final session reviewed the changes and their potential generalizations in the future, as well as empowerment strategies used by participants. The four nurses who led the intervention had more than 20 years of experience in psychiatry and mental health and completed 2 days of specific training. They received one-hour supervision sessions for each participant from experienced clinical nurses to ensure the fidelity of the intervention delivery (20).

#### 2.7.3 Intervention delivery

The Ensemble program was administered in addition to SAU. No attempts were made to standardize SAU treatment and participants were evaluated for SAU at baseline. Among them, 21 (26.3%) participants in the control group and 19 (23.8%) participants in the Ensemble group did not report any ongoing supportive intervention. Regarding the 21 participants without any support in the SAU condition, intervention providers oriented 10 participants toward the intervention and provided telephonic support to one participant because of poor health and pandemic-related conditions while waiting for the active Ensemble program.

### 2.8 Primary outcomes

The primary outcomes included three global distress indices—GSI, Positive Symptom Distress Index (PSDI), and Positive Symptom Total (PST)—specifically examining changes between T0–T1 and T0–T2 for GSI on the BSI (21).

### 2.9 Secondary outcomes

Secondary outcomes included changes in scores from baseline to post-intervention and follow-up for informal caregivers’ burden, measured by the ZBI, which included 22 items (23); caregivers’ optimism levels, measured by the LOT-R, which included 10 items (22); and quality of life, measured by the SF-36, which included 36 items, with a specific focus on the mental health score, which was composed of four dimensions: emotional well-being, social functioning, energy/fatigue, and role limitations due to emotional problems (24).

### 2.10 Statistical methods

A mixed models for repeated measures (MMRM) analysis of variance was used to test group differences in improvement in mental health and burden over time at the two endpoints. Time was introduced as a within-group factor, and intervention as a between-group factor. The model’s main effects of intervention and time, as well as their interactions, were examined. Planned comparisons within the MMRM were performed to examine contrasting changes from the first assessment to different endpoints, considering the numerous measurements available. This enabled the testing of between-group differences in the improvement rate in scores from the first assessment to various endpoints. Analyses were conducted based on an intent-to-treat approach to assess clinical benefits for psychological health and burden levels and to maintain the advantages of randomization (27).

The Akaike information criterion (AIC) coefficient was used to determine the optimal within-subject covariance matrix. Unstructured, autoregressive, compound symmetric, Toeplitz structures and their heterogeneous versions were tested. Statistical analyses were performed using IBM-SPPS Version 25. All statistical tests were two-tailed, and significance was determined at the 0.05 level.

Concerning multiple secondary outcomes, multiple comparisons can sometimes be used as justification for employing “corrected” *p*-values, using methods such as Bonferroni correction (28). However, in this study, corrections for separate hypothesis-driven tests were not indicated because these corrections involve comparing each hypothesis to a universal null hypothesis, predicting no difference across all areas of the different tests performed (29). Since these corrections were omitted, the risk of Type I errors must be considered when interpreting the identified associations.

Overall, the collected data were of high quality. In the case of any missing responses, they were replaced with the average of the participants’ items, given that more than 50% of the subscale had been completed.

## 3 Results

### 3.1 Participant recruitment and follow-up information

**Figure 2:**
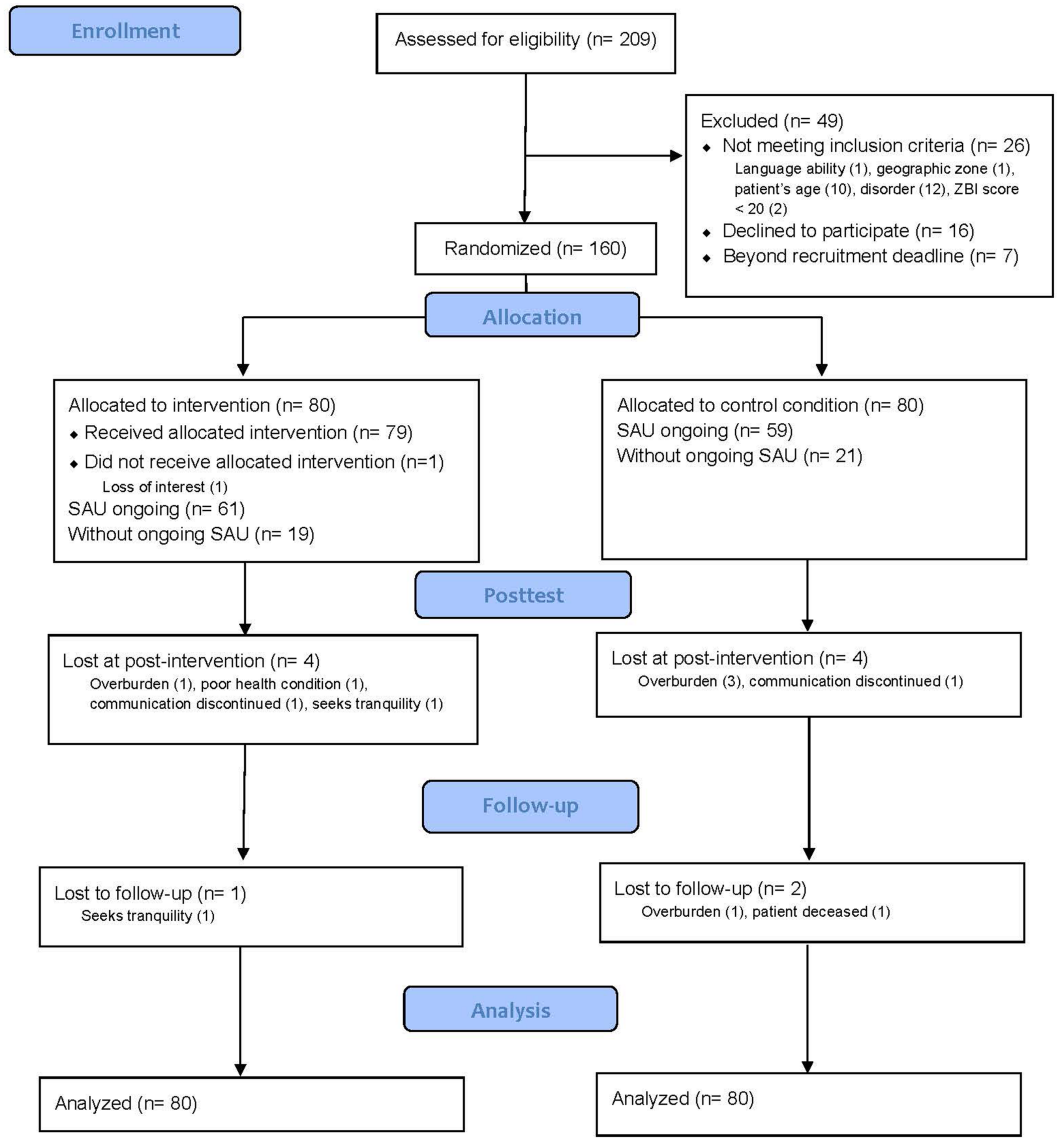
CONSORT flow diagram.

A total of 209 participants were invited to participate in the trial between October 2019 and August 2022. The final evaluation was conducted in January 2023. Before randomization, 49 caregivers were excluded for various reasons: 16 declined to participate, 26 did not meet the inclusion criteria, and 7 reached out to the research team after the recruitment deadline. Of the initial 160 participants randomized at baseline, 8 individuals (4 from the Ensemble group and 4 from the SAU group) dropped out between the first and second evaluations. At the 2-month follow-up, an additional participant from the Ensemble group and two from the control group dropped out, resulting in a total of 149 participants, which represented 93.12% of the initial sample. At T1, six participants disclosed their allocations. To maintain rapport, the same research assistant conducted the T2 assessment because of the nature of auto-reported evaluations. Further details on recruitment are reported in Figure 2 according to the CONSORT flowchart.

### 3.2 Sociodemographic and baseline clinical data

The average age of the informal caregivers was 52 years (SD = 13.45). The majority were women (79.4%). Eighty-six caregivers were married or cohabiting (53.8%), and 90 had a university-level education (56.3%). For the entire sample, professional activities were divided into three comparable levels, with 56 caregivers (35.4%) working full-time, 55 (34.8%) working part-time, and 47 (29.7%) unemployed or retired. Ninety-two caregivers were parents of the patient (57.5%), and 33 were their partners (20.6%). Ninety-nine caregivers (61.9%) had daily contact with the patient, and 82 lived with the patient (51.2%).

Ninety-eight of the patients receiving care were male (61.3%), with a mean age of 37.20 (SD = 15.42). The primary diagnosis, according to the International Classification of Diseases 11th Revision (ICD-11), was schizophrenia or other primary psychotic disorders (70, 43.8%). The mean duration of mental illness was 13.01 years (SD = 11.94). Mean SOFAS scores assessed at baseline were similar. For the entire sample, the mean score for item 1 (current social function) was 43.09 (SD = 18.78); it was 44.07 (SD = 18.59) in the Ensemble group and 42.11 (SD = 19.04) in the SAU group. For item 2 (social function before illness), the mean scores were 80.50 (SD = 17.23), 80.28 (SD = 15.86), and 80.72 (SD = 18.61), for the entire sample, Ensemble group, and SAU group, respectively. Additional sociodemographic and clinical data are presented in Table 1.

**Table 1:** Sociodemographic and clinical data by group.

	Total	Ensemble	SAU
	*N* = 160	*N* = 80	*N* = 80
**Age in years, M (SD)**	52.29 (13.455)	51.28 (13.703)	53.31 (13.210)
**Gender, male % (*n*)**	20.6 (33)	15.0 (12)	26.3 (21)
Marital status, % (*n*)			
Single	20.0 (32)	21.3 (17)	18.8 (15)
Separated	6.3 (10)	5.0 (4)	7.5 (6)
Divorced	15.0 (24)	17.5 (14)	12.5 (10)
Married/Cohabiting	53.8 (86)	50.0 (40)	57.5 (46)
Widower	5.0 (8)	6.3 (5)	3.8 (3)
Level of education, % (*n*)			
Compulsory education completed	1.9 (3)	1.3 (1)	2.5 (2)
Apprenticeship of 3 years or more	17.5 (28)	21.3 (17)	13.8 (11)
Federal maturity or baccalaureate	8.8 (14)	10.0 (8)	7.5 (6)
Higher education (professional, commercial, technical)	15.6 (25)	13.8 (11)	17.5 (14)
Universities, universities of applied sciences	56.3 (90)	53.8 (43)	58.8 (47)
Professional activity, % (*n*)^a^			
Full-time job	35.4 (56)	34.2 (27)	36.7 (29)
Part-time job	34.8 (55)	30.4 (24)	39.2 (31)
Unemployed or retired	29.7 (47)	35.4 (28)	24.1 (19)
Type of relationship with the person with mental illness, % (*n*)			
Parent	57.5 (92)	57.5 (46)	57.5 (46)
Child	10.6 (17)	13.8 (11)	7.5 (6)
Partner	20.6 (33)	16.3 (13)	25.0 (20)
Sibling	7.5 (12)	7.5 (6)	7.5 (6)
Other family member	1.3 (2)	1.3 (1)	1.3 (1)
Friend	1.3 (2)	1.3 (1)	1.3 (1)
Other	1.3 (2)	2.5 (2)	0 (0)
**Age of the person with mental illness in years, M (SD)**	37.20 (15.424)	37.20 (15.102)	37.20 (15.836)
**Gender of the person with mental illness, male, %(*n*)**	61.3 (98)	60.0 (48)	62.5 (50)
**Joint household with the person with mental illness, % (*n*)**	51.2 (82)	47.5 (38)	55 (44)
Frequency of contact with the person with mental illness, % (*n*)			
No contact	1.9 (3)	2.5 (2)	1.3 (1)
≥ 1 h once a month	5.6 (9)	7.5 (6)	3.8 (3)
1 to 3 times a week	18.1 (29)	18.8 (15)	17.5 (14)
4 to 6 times a week	12.5 (20)	13.8 (11)	11.3 (9)
Daily (no joint household)	16.3 (26)	16.3 (13)	16.3 (13)
Daily (joint household)	45.6 (73)	41.3 (33)	50.0 (40)
Diagnosis of the person with mental illness ICD-11, % (*n*)			
Schizophrenia or other primary psychotic disorders	43.8 (70)	48.8 (39)	38.8 (31)
Bipolar disorder	17.5 (28)	17.5 (14)	17.5 (14)
Depressive disorders	17.5 (28)	11.3 (9)	23.8 (19)
Anxiety or fear-related disorders	2.5 (4)	1.3 (1)	3.8 (3)
Disorders specifically associated with stress	3.8 (6)	6.3 (5)	1.3 (1)
Dissociative disorders	1.3 (2)	0.0 (0)	2.5 (2)
Feeding or eating disorders	0.6 (1)	1.3 (1)	0.0 (0)
Disorders due to substance use	1.9 (3)	2.5 (2)	1.3 (1)
Personality disorders	9.4 (15)	10.0 (8)	8.8 (7)
Other	1.9 (3)	1.3 (1)	2.5 (2)
**Duration of mental illness in years, M (SD)**	13.01 (11.942)	12.825 (12.498)	13.194 (11.436)
Ongoing support, % (*n*)			
Professional support	51.2 (82)	55.0 (44)	47.5 (38)
Associative support	16.3 (26)	15.0 (12)	17.5 (14)
Personal resources	7.5 (12)	6.3 (5)	8.8 (7)
None	25.0 (40)	23.8 (19)	26.3 (21)
**SOFAS 1 (actual), M (SD)**	43.09 (18.788)	44.07 (18.595)	42.11 (19.044)
**SOFAS 2 (past), M (SD)**	80.50 (17.233)	80.28 (15.856)	80.72 (18.605)

^a^2 missing; SOFAS, Social and Occupational Functioning Assessment Scale; SAU, support as usual; ICD-11, International Classification of Diseases 11th Revision.

### 3.3 Primary outcomes

The primary outcomes included the three global distress indices on the BSI: the GSI, PSDI, and PST. Contrary to the first hypothesis, the MMRM analysis (Table 2) for the primary outcome, GSI, showed no significant effect at the two endpoints in favor of the Ensemble group (T1: *t*(153.83) = −0.97, *p* = 0.333, T2: *t*(154.24) = −1.72, *p* = 0.088). The PSDI difference was not significant at posttest (T1: *t*(208.21) = −1.49, *p* = 0.137) but was significant at the two-month follow-up (T2: *t*(147.79) = −2.07, *p* = 0.041). No significant difference was found in the PST changes (ΔT0-T1: *t*(158.00) = −0.23, *p* = 0.820, ΔT0-T2: *t*(158.00) = −1.13, *p* = 0.262).

**Table 2:** Analyses of outcome measures for T0–T1–T2.

	Marginal means – estimates (SE)											
	Ensemble program + SAU			SAU			Planned comparison 1 (T0–T1)			Planned comparison 2 (T0–T2)		
	T0	T1	T2	T0	T1	T2	Estimate (SE)	t(*df*)	p	Estimate (SE)	t(*df*)	p
Main outcomes												
BSI Global severity index	0.858 (0.059)	0.615 (0.048)	0.578 (0.052)	0.764 (0.059)	0.580 (0.048)	0.600 (0.052)	−0.595 (0.061)	t(153.834) = −0.971	0.333	−0.117 (0.680)	t(154.240) = −1.718	0.088
Positive symptom total	24.750 (1.224)	22.862 (1.441)	22.175 (1.504)	22.950 (1.224)	21.462 (1.441)	22.762 (1.504)	−0.400 (1.758)	t(158.000) = −0.228	0.820	−2.388 (2.122)	t(158.000) = −1.125	0.262
BSI Positive symptom distress index	1.715 (0.051)	1.446 (0.042)	1.406 (0.047)	1.631 (0.051)	1.449 (0.042)	1.459 (0.048)	−0.087 (0.058)	t(208.215) = −1.494	0.137	−0.138 (0.067)	t(147.793) = −2.067	0.041*
Secondary outcomes												
ZBI	48.426 (1.395)	39.578 (1.628)	39.316 (1.726)	46.750 (1.395)	42.933 (1.628)	40.477 (1.733)	−5.031 (1.576)	t(151.501) = −3.192	0.002**	−2.838 (1.809)	t(151.146) = −1.568	0.119
LOT-R	15.400 (0.490)	16.198 (0.495)	16.227 (0.497)	15.913 (0.490)	15.575 (0.496)	15.654 (0.498)	1.135 (0.438)	t(262.662) = 2.594	0.010**	1.086 (0.495)	t(149.553) = 2.194	0.030*
SF-36 Mental general score	209.827 (5.902)	214.304 (6.003)	225.612 (6.043)	201.183 (5.902)	216.304 (6.025)	216.957 (6.068)	−9.986 (7.765)	t(265.124) = −1.286	0.200	0.011 (8.566)	t(168.043) = 0.001	0.999
Exploratory analyses on BSI dimensions												
BSI Somatization	0.764 (0.078)	0.567 (0.061)	0.551 (0.067)	0.657 (0.078)	0.480 (0.061)	0.486 (0.067)	−0.203 (0.831)	t(153.879) = −0.244	0.807	−0.042 (0.089)	t(153.846) = −0.467	0.641
BSI Obsession-compulsion	1.117 (0.095)	0.843 (0.082)	0.843 (0.086)	1.098 (0.095)	0.941 (0.082)	0.968 (0.086)	−0.177 (0.097)	t(153.735) = −1.818	0.071	−0.204 (0.112)	t(154.148) = −1.826	0.070
BSI Interpersonal sensitivity	0.859 (0.084)	0.604 (0.071)	0.468 (0.070)	0.766 (0.084)	0.581 (0.071)	0.581 (0.070)	−0.071 (0.102)	t(241.421) = −0.698	0.486	−0.206 (0.102)	t(241.604) = −2.032	0.043*
BSI Depression	0.931 (0.087)	0.688 (0.070)	0.613 (0.080)	0.925 (0.087)	0.695 (0.070)	0.730 (0.081)	−0.013 (0.095)	t(210.665) = −0.134	0.894	−0.123 (0.112)	t(154.842) = −1.102	0.272
BSI Anxiety	0.962 (0.082)	0.632 (0.064)	0.639 (0.074)	0.896 (0.082)	0.588 (0.064)	0.628 (0.074)	−0.023 (0.092)	t(204.001) = −0.253	0.801	−0.055 (0.106)	t(151.491) = −0.519	0.605
BSI Hostility	0.925 (0.069)	0.713 (0.058)	0.626 (0.059)	0.835 (0.069)	0.581 (0.058)	0.663 (0.059)	0.042 (0.077)	t(214.530) = 0.547	0.585	−0.126 (0.085)	t(152.943) = −1.492	0.138
BSI Phobic anxiety	0.400 (0.056)	0.287 (0.052)	0.263 (0.052)	0.260 (0.056)	0.244 (0.052)	0.216 (0.052)	−0.096 (0.081)	t(156.839) = −1.183	0.239	−0.093 (0.083)	t(156.124) = −1.129	0.261
BSI Paranoid ideation	0.830 (0.070)	0.583 (0.066)	0.508 (0.063)	0.668 (0.070)	0.542 (0.066)	0.563 (0.063)	−0.122 (0.085)	t(154.434) = −1.434	0.153	−0.217 (0.086)	t(155.332) = −2.518	0.013**
BSI Psychoticism	0.593 (0.059)	0.402 (0.046)	0.393 (0.049)	0.488 (0.059)	0.380 (0.046)	0.371 (0.050)	−0.084 (0.069)	t(207.975) = −1.218	0.224	−0.083 (0.077)	t(156.968) = −1.082	0.281

* *p*-value < 0.05. SAU, support as usual; T0, pretest; T1, posttest at 2 months; T2, follow-up at 4 months; BSI, Brief Symptom Inventory; ZBI, Zarit Burden Interview; LOT-R, Revised Life Orientation Test; SF-36, 36-Item Short Form Health Survey; SE, standard error.

### 3.4 Secondary outcomes

The secondary outcomes included ZBI, LOT-R, and SF-36 mental component scores. The MMRM analysis showed a significant reduction in burden in the Ensemble group on the ZBI post-intervention (*t*(151.50) = −3.19, *p* = 0.002). The results also showed a significative increase in optimism levels on the LOT-R for the Ensemble group at both endpoints (ΔT0-T1: *t*(262.66) = 2.59, *p* = 0.010, ΔT0-T2: *t*(149.55) = 2.19, *p* = 0.030). However, post-intervention, there was no significant difference in the mental component score on the SF-36 between the groups (*t*(265.12) = −1.29, *p* = 0.200).

### 3.5 Exploratory analyses

In addition to the three global distress indices, the BSI provides nine subscales for symptomatic conditions. Exploratory analyses of these subscales were performed to better understand the effects of the interventions (Table 3). The results indicated a significant decrease in interpersonal sensitivity (*t*(241.60) = −2.03, *p* = 0.043) and paranoid ideation (*t*(155.33) = −2.52, *p* = 0.013) at the two-month follow-up. No significant differences were observed in the remaining seven subscales.

**Table 3:** Clinical changes on the GSI and ZBI.

		Ensemble, % (*n* = 80)	SAU, % (*n* = 80)
**GSI**			
	Improved	41.3 (33)	37.5 (30)
	Unchanged	46.3 (37)	47.5 (38)
	Worsened	7.5 (6)	10.0 (8)
**ZBI**			
	Improved	47.5 (38)	18.8 (15)
	Unchanged	43.8 (35)	72.5 (58)
	Worsened	3.8 (3)	3.8 (3)

The cut-off value was based on the standard error of the difference and an α-level of 0.05. GSI, Global Severity Index; ZBI, Zarit Burden Interview.

### 3.6 Comparisons of clinical changes in outcomes

Clinical changes in participants’ post-intervention GSI and ZBI scores are shown in Table 3. Cut-off scores were estimated using a standard error of the difference with an alpha level of 0.05. In the SAU group, 30 participants (37.5%) demonstrated significantly improved scores on the GSI, 38 (47.5%) had unchanged scores, and 8 (10.0%) exhibited deteriorated scores. In the Ensemble group, 33 participants (41.3%) showed improvement, 37 (46.3%) had similar scores, and 6 (7.5%) demonstrated worsened scores. Regarding changes in the ZBI, 15 participants (18.8%) improved in the SAU group, 58 (72.5%) remained similar, and 3 (3.8%) experienced a decline. In the Ensemble group, 38 (47.5%) participants improved, 35 (43.8%) had unchanged scores, and 3 (3.8%) experienced worsened scores.

## 4 Discussion

The current RCT aimed to assess the efficacy of the Ensemble program, a tailored intervention designed to support informal caregivers of adults with severe mental disorders. The results showed that the Ensemble program led to a significant improvement in the Positive Symptom Distress Index of the BSI 2 months after the completion of the intervention, but not the BSI global index, as predicted in the pilot study. The BSI was used to identify self-reported clinically relevant psychological symptoms. Improving informal caregivers’ psychological health status presents a significant clinical challenge because they are not typically considered care recipients, and existing data on informal caregivers have shown that they often suffer from psychological symptoms (30, 31). However, the GSI, an index providing a global indicator of psychological distress, showed a tendency to be significant over time (*p* = 0.088 at T2). Nevertheless, we cannot rule out that its effect may have been overestimated in the pilot study, and, therefore, more sensitive outcomes, such as anxiety or depression, need to be considered (31). Systematic literature reviews regarding informal caregivers’ health status highlight the inconsistency in results and the challenges in improving their health (30, 32).

The burden reduction associated with the Ensemble program at post-intervention highlights the instant effectiveness of the intervention on this secondary outcome. The positive impact observed immediately after program completion is encouraging because burden reduction improves informal caregivers’ coping strategies and their ability to support patients in their recovery process (8). By preventing stress factors linked to the patient’s illness and the role implications of the informal caregiver, improved experiences and adaptability in caring for individuals suffering from severe mental illness can be facilitated. When informal caregivers derive more satisfaction in the outcomes of their involvement and associate helpful meaning with this experience, family interactions, as well as the relationship with the affected relative, may improve (33). For example, existing literature suggests that social support, primarily offered by the family, is known to reduce schizophrenia relapses (34).

The outcomes of this study also indicate a significant improvement in optimism levels. An improved level of optimism may likely denote a personal coping process that could lead to more active involvement in the caregiving role while considering one’s own abilities and well-being (4). The reduction in burden and improvement in optimism offer an opportunity to harmonize efforts and resources to improve patient support without compromising the well-being of caregivers (35). In addition, the effects observed on the dimension scales, such as interpersonal sensitivity (e.g., feeling self-conscious around others) and paranoid ideation (e.g., attributing most of your troubles to others), are encouraging. Similar results for paranoid ideation were reported in the pilot phase (2). Significant effects on paranoid ideation can be correlated to a better understanding of mental health disorders, reducing inappropriate attribution of the disease and empowering individuals with the notion of recovery. A more appropriate attribution of the disorder allows for better acceptance and adaptiveness in coping strategies, improving the supportive role (13,34). In addition, improving interpersonal skills can have a positive impact on collaborative work with healthcare staff (36).

Given that informal caregivers are key stakeholders within the healthcare system as a result of their informal and unpaid support of patients, integrated care, including family intervention, is essential for promoting the quality of psychosocial care (37). Although their role has been recognized in the literature, studies examining the economic benefits of supporting informal caregivers are still lacking (38).

### 4.1 Strengths and limitations

One significant strength of this study, as compared to other studies in the field (7, 11), is the inclusion of all caregivers who experienced a high level of burden. Clinical research cannot continue to exclude relatives from interventions focusing on them. Traditional group sessions often exclude relatives who cannot attend weekly sessions, those who have previously undergone psychotherapy or psychoeducation, and those dealing with undiagnosed patients. Conversely, this study included vulnerable caregivers, who are often excluded from evidence-based interventions (8).

Another strength of the study lies in the fact that the intervention providers were mobile and met participants in the consultation setting, at home, or in local association offices. This approach supported the participants’ needs, regardless of the presence of local support. However, this flexibility could also be seen as a limitation as mobile mental health teams remain scarce. Regardless of the financial constraints linked to the location of the Ensemble program, this study demonstrated that it was suitable for all care settings.

The improvements observed in the control group can partly be explained by the research evaluation sessions. Each participant had the opportunity to discuss their current situation with the assessor across all three sessions, facilitating a trusting relationship that particularly helped isolated informal caregivers without any support. Since the assessors were nurses with psychiatric experience, this likely fostered a supportive environment. Though the specific effectiveness of the Ensemble program cannot be accurately assessed due to the absence of an active, homogeneous control group, our results provide a foundation for testing this effectiveness in future research. Another limitation of this study is its short time span: Two months of follow-up is a short period for informal caregivers who will be caring for their patients for longer periods. Thus, future studies should establish longer follow-up periods.

## 5 Conclusion

Current trends suggest that tailored brief informal caregiver-focused interventions are recommended to respond to the multiple and varied needs of individuals with high levels of burden. In response to a lack of scientific recommendations stemming from the inconsistency of content related to interventions for informal caregivers who face a high care burden (8), this study supports the Ensemble program as a suitable intervention. Although the healthcare system is struggling to meet the needs of this population, there is a growing demand for relevant clinical alternatives to avoid increasing healthcare costs. Future studies could assess not only health-related outcomes but also accessibility to services in terms of user satisfaction and hospitality.

## Data availability statement

The raw data supporting the conclusions of this article will be made available by the authors, without undue reservation.

## Ethics statement

The studies involving humans were approved by the Human Research Ethics Committee of Vaud State, Switzerland, approved the research protocol (Project ID-2019-01181). The studies were conducted in accordance with the local legislation and institutional requirements. The participants provided their written informed consent to participate in this study.

## Author contributions

SR: Conceptualization, Data curation, Formal analysis, Funding acquisition, Methodology, Project administration, Resources, Software, Supervision, Validation, Writing – original draft, Writing – review & editing. DM: Conceptualization, Data curation, Formal analysis, Methodology, Validation, Writing – original draft, Writing – review & editing. PG: Conceptualization, Formal analysis, Methodology, Validation, Writing – review & editing. CC-T: Investigation, Methodology, Validation, Writing – review & editing. SM: Investigation, Validation, Writing – review & editing. LB: Investigation, Validation, Writing – review & editing. A-LD: Investigation, Validation, Writing – review & editing. CB: Validation, Writing – review & editing. AI: Resources, Validation, Writing – review & editing. AN: Resources, Validation, Writing – review & editing. JF: Conceptualization, Formal analysis, Funding acquisition, Methodology, Supervision, Validation, Writing – original draft, Writing – review & editing.
